# Regulation of the Ocular Cell/Tissue Response by Implantable Biomaterials and Drug Delivery Systems †

**DOI:** 10.3390/bioengineering7030065

**Published:** 2020-06-30

**Authors:** Francesco Baino, Saeid Kargozar

**Affiliations:** 1 Department of Applied Science and Technology, Institute of Materials Physics and Engineering, Politecnico di Torino, 10129 Turin, Italy; 2 Tissue Engineering Research Group (TERG), Department of Anatomy and Cell Biology, School of Medicine, Mashhad University of Medical Sciences, Mashhad 917794-8564, Iran; kargozarsaeid@gmail.com

**Keywords:** ocular biomaterials, drug delivery, polymers, cell-biomaterial interaction

## Abstract

Therapeutic advancements in the treatment of various ocular diseases is often linked to the development of efficient drug delivery systems (DDSs), which would allow a sustained release while maintaining therapeutic drug levels in the target tissues. In this way, ocular tissue/cell response can be properly modulated and designed in order to produce a therapeutic effect. An ideal ocular DDS should encapsulate and release the appropriate drug concentration to the target tissue (therapeutic but non-toxic level) while preserving drug functionality. Furthermore, a constant release is usually preferred, keeping the initial burst to a minimum. Different materials are used, modified, and combined in order to achieve a sustained drug release in both the anterior and posterior segments of the eye. After giving a picture of the different strategies adopted for ocular drug release, this review article provides an overview of the biomaterials that are used as drug carriers in the eye, including micro- and nanospheres, liposomes, hydrogels, and multi-material implants; the advantages and limitations of these DDSs are discussed in reference to the major ocular applications.

## 1. Introduction

Ocular diseases can be induced by a number of factors and affect both the anterior and the posterior segment of the eye. The most common pathologies of the anterior segment are inflammatory diseases such as blepharitis, conjunctivitis, dry eye syndrome, and uveitis [1]. Diseases of the posterior segment typically comprise some chronic pathologies, the incidence of which significantly increased over recent years due to the increase of the average life expectancy [1]. These pathologies include choroidal neovascularization (CNV) following age-related macular disease (AMD), diabetic retinopathy retinal vein occlusion, macular edema, and glaucoma. When left untreated, these diseases of the posterior segment can lead to severe visual complications including dramatic visual deficiency and even blindness, with an obvious impact on the patient’s quality of life and additional costs for society. It was estimated that, in the early 2000s, there were 160 million people suffering from an important visual deficiency worldwide and 37 million totally blind patients [2]. A more recent study published in 2015 reported that there were 216 million people having a moderate to severe visual deficiency and 12.9 to 65.4 million blind patients [3]. Projections for 2020 indicate an increase to 237 million and 70.9 million people suffering from partial and complete blindness, respectively [3].

In most cases, the treatment of chronic ocular diseases require periodic application of drugs. The frequency, duration, and route of application of these drug-based treatments vary depending on the specifics of the disease, as well as the pharmacological and pharmacokinetic properties of the molecule being delivered. For example, the diseases of the posterior segment like AMD can be treated by intravitreal injections. Before 1999, fewer than 3000 intravitreal injections were delivered in the United States annually; this number grew to one million in 2008 and over four million in 2013, rising further with an increased trend [3]. This witnesses how the field of ocular drug release is significant in modern society and clinics. 

The eye offers multiple entry routes through which ocular drugs may be delivered [4,5,6,7]. The topical route is the most common route of drug administration for the ocular surface and anterior segment including cornea, conjunctiva, and anterior chamber [8]. This involves the easy self-application of drug-containing solutions/suspension; however, it was estimated that less than 5% of drug can actually reach the anterior segment due to the resistive properties of corneal barrier [9]. Therefore, there is also the possibility of injecting the drug through into the subconjunctival space or directly inside the anterior chamber in order to increase the drug concentration in the target organ.

The topical route can also be used to treat posterior segment diseases, although other strategies are typically preferred and more efficient, including systemic, periocular (injections that are carried out in the periocular area under the Tenon’s capsule), and intravitreal (within the vitreous) administration. The latter two are the most commonly used clinically.

The therapeutic efficacy of drugs is strongly related to the pharmacological characteristics of the molecule being delivered, as well as to the route of administration and the limiting barriers to drug perfusion at the tissue level. Each delivery route has its own advantages and disadvantages. For example, the topical route has the advantage of avoiding a direct contact/physical intervention on the eyeball and is an easy strategy to treat various ocular disorders needing repetitive administration, such as glaucoma, which is the first cause of irreversible blindness worldwide and needs chronic treatment [10]. Glaucoma therapy is mainly topical by means of ophthalmic solutions to be instilled daily in the affected eye for very long periods of time, even for a lifetime. However, the therapeutic efficacy of the topical administration route almost entirely depends on the patient’s co-operation, which may be suboptimal and difficult to control, and the drug can be lost by drainage of the instilled solution through tears. In order to overcome these problems, miniaturized implants were recently proposed for aqueous humor drainage and local release of hypotonic drugs.

In the attempt of providing feasible and more effective alternatives to many of the current therapies for the anterior and posterior segment of the eye, new biomaterials and systems were developed such as nano-emulsions, hydrogels, and vesicular systems (liposomes) [11,12,13,14,15,16]. These biomaterials typically belong to the class of polymers, which are soft and well-tolerated by ocular tissues in a number of surgical and non-surgical applications [17,18,19,20]. Controlled drug delivery systems (DDSs) are very appropriate in those cases where a repeated dosing or injection is required. The goal of DDSs is to increase periods of ocular drug contact and overall drug delivery by bypassing the ocular tissues that act as limiting barriers to drug perfusion [21,22]. The targeted and prolonged release of ocular drugs is a rapidly evolving field due to the advent of new biomaterials that are being developed in a context of continuous research making impressive progress in recent years.

## 2. A Short Overview of Eye Anatomy

The eye is an organ of great complexity. It plays a key role in the visual process as it is the receptor of optical information that is then processed by the retina and transmitted to the brain by means of the optic nerve [17].

An average adult human eye is roughly spherical and measures approximately 24 mm in diameter. The eye lies in the cavity of the bony orbit, which acts as a mechanical protector preventing trauma, and it is anteriorly covered by the eyelids that are responsible for producing and distributing tears over the eyeball surface while regulating the amount of light accessing the eye.

**Figure 1:**
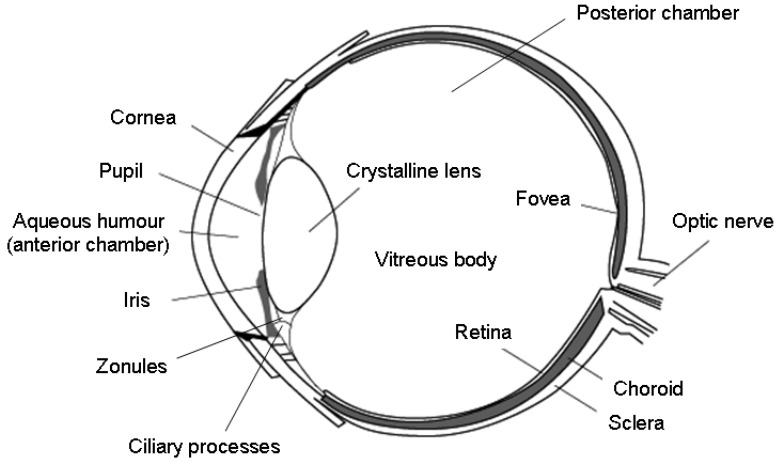
Schematic illustration of the eye anatomy. Reproduced from Reference [17].

The eyeball consists of three tunics of distinct structure (Figure 1): (i) the outermost white fibrous membrane is the sclera, with its clear anterior part, the cornea, which acts as the eye’s outermost lens; (ii) the middle vascular tunic, which encompasses the choroid, the iris, and the ciliary body; (iii) the interior nervous membrane and the retina. There are two fluid media within the eyeball: the aqueous humor and the vitreous body. These are separated by the crystalline lens and its suspensory ligaments. The aqueous humor is a transparent fluid secreted by the ciliary processes into the anterior and posterior chambers, and it provides nutrition to the areas lacking blood vessels (i.e., the lens and the cornea). The vitreous body is a gelatinous substance that gives the eye structural support and fills the space between the lens and the retina, which processes the light through a layer of photoreceptor cells. The eye is schematically illustrated in Figure 1, and a concise description of its main components and relative functions is provided in Table 1.

Each eye has six extraocular muscles (EOMs), functioning as antagonistic pairs. These muscles are responsible for holding the eye steady or moving it along three different axes: horizontal, either toward the nose (adduction) or away from the nose (abduction); vertical, either upward or downward; and torsional—movements that rotate the top of the eye toward the nose (intorsion) or away from the nose (extorsion). The EOMs originate in the posterior orbit, are attached to the sclera, and are enveloped together with the eyeball and the optic nerve by the Tenon’s capsule to form a cone-shaped unit within the bony orbit. Tenon’s capsule aids in suspending the orbital structures and acts as an extraocular muscle pulley.

## 3. Strategies of Ocular Drug Administration

Various strategies can be applied to provide a pharmacological therapy to ocular tissues and structures. The routes of administration of drugs to the anterior or posterior segments of the eye are topical, intracameral (in the anterior chamber), transcleral/periocular, intravitreal, suprachoroidal (seldom), and systemic.

### 3.1. Topical Route

The topical route is the most commonly used approach in current clinical practice, both in hospital and in home therapy, due to its ease and non-invasiveness. The drug is provided in the form of eye drops or ointment that can be useful in the treatment of many disorders of the ocular surface and anterior segment.

Topical treatments used for anterior segment diseases must first pass through the corneal epithelium, which exhibits a significant “barrier effect” to the penetration of drug molecules into the tissue due to its hydrophobic characteristics [23]. Specifically, the corneal epithelium is lipophilic and is the main barrier to the passage of hydrophilic drugs, while the hydrophilic stroma constitutes a barrier to lipophilic substances. Another limiting factor for topical administration is the molecular weight of the drug as it was estimated that only small molecules (less than 1 kDa) can easily pass through the corneal endothelium [24].

Under normal conditions, a human eye can host 25–30 μL of an ophthalmic solution; further reductions in the amount that is absorbed are due to blinking, i.e., the drainage of tears through the outflow pathways which causes systemic absorption via the nasal mucosa and the gastrointestinal tract [25].

In order to improve topical absorption and, hence, the bioavailability of drugs in intraocular tissues, research is ongoing to find suitable drug carriers that increase the time of contact with the ocular surface and/or the passage of drugs through ocular barriers [26,27]. It is interesting to point out that only 1–10% of the instilled dose of drug is absorbed at the ocular level and only about 1% reaches the aqueous humor [28].

### 3.2. Intracameral Route

Intracameral injection provides high drug concentrations in the anterior chamber. This route is commonly used for the anesthesia of the eyeball, the prevention of endophthalmitis during ocular surgery (especially cataract), and the treatment of inflammations of the anterior segment [29]. The most widely used antibiotics in such a strategy are vancomycin, moxifloxacin, and cephalosporins. It was previously shown that intracameral delivery of dexamethasone may result in a decrease in skin hypopigmentation, extraocular muscle atrophy, and subdermal fat atrophy [30]. However, some drawbacks have to be accounted for this route including toxic anterior segment syndrome (TASS), which is a consequence of introducing a noninfectious substance to the anterior segment [31].

### 3.3. Transcleral/Periocular Route

The sclera has a fibrous structure and exhibits a large surface (about 95% of the total surface of the eye) which offers less resistance to the diffusion of substances compared to the cornea [32]. The transcleral/periocular approach takes advantage of this feature and allows obtaining a greater permeation of the drug. However, this administration route is recommended only for drugs that have a molecular weight lower than about 70 kDa.

### 3.4. Intravitreal Route

Intravitreal injections are the most invasive way to obtain therapeutic concentrations of the drug in the posterior segment. For this reason, serious complications are possible, including cataracts, vitreous hemorrhage, retinal detachment, and endophthalmitis [33]. Surgical care and the use of accurate sterilization protocols minimize the incidence of such complications. This administration route is used in the therapy of exudative senile macular degeneration, where anti-vascular endothelial growth factor (VEGF) drugs (e.g., bevacizumab, ranibizumab, and aflibercept) are injected every 30–60 days to reach and maintain a sustained therapeutic concentration [34]. The high cost and obvious impact of this strategy on the patient led to the development of minimally invasive DDSs that are positioned inside the eyeball in order to reduce the frequency of intravitreal injections.

Apart from being used to supply anti-VEGF agents, the intravitreal injection method typically provides corticosteroids and antibiotics; intravitreal solid implants for long-term drug release include Ozurdex (Allergan, Irvine, CA, USA) and Illuvien (Alimera Sciences, Alpharetta, GA, USA).

### 3.5. Sub-Retinal Route

The sub-retinal space provides a good space for injection of different drugs and therapeutic substances (e.g., gene therapy, cell therapy) to treat vitreoretinal diseases [35]. In this route, drugs administrated are in direct contact with the plasma membrane of the photoreceptors, retinal pigment epithelium (RPE), and subretinal blebs [36,37]. Due to its high precision and efficacy, the sub-retinal route is considered as one of the most effective methods used for ocular drug delivery [38]. The sub-retinal space can be typically accessed by the posterior trans-scleral route or trans-choroidal route (passing through the choroid and Bruch’s membrane without penetrating the retina) [39,40,41,42,43,44].

### 3.6. Suprachoroidal Route

This strategy is relatively less common as compared to the other approaches. In a recent study, Lewis et al. [45] developed an iTrack microcatheter system through which drug molecules, such as triamcinolone acetonide (TA) and bevacizumab, can be released into the suprachoroidal space. Patel et al. [46] in an experimental study on animal eyes used borosilicate microneedles to inject boluses of drugs, as well as micro- and nanospheres, into the suprachoroidal space. Suprachoroidal microneedles releasing triamcinolone acetonide are currently being studied to treat uveitis [47].

### 3.7. Systemic Route

This strategy may be applied when the other ones are discouraged or unfeasible due to the specific clinical case, or when the other approaches would imply the local administration of too high doses of drug, thus carrying the risk of toxic effects [48]. However, systemically administered drugs should pass the blood–aqueous barrier at the level of the anterior segment and the blood–retinal barrier at the level of the posterior segment. The latter has tight junctions that do not allow several drug molecules to enter the posterior segment. Therefore, therapeutic concentrations of drugs in the posterior segment are better obtained locally rather than through systemic administration [49]. Furthermore, systemic drug delivery may be associated with immunological and metabolic side effects [50].

## 4. Biomaterials and Implants for the Ocular Release of Therapeutics

Over the last few years, a number of implantable systems were designed and tailored to release therapeutics into the eye to elicit an appropriate response by ocular tissues. Organic biomolecules (e.g., antibiotics, anti-inflammatory, or anti-proliferative drugs) and, more rarely, therapeutic metallic cations were experimented and/or clinically tested for this purpose.

### 4.1. Therapeutic Ion Release from Orbital Implants and Ocular Prostheses

Orbital implants are introduced into the patient’s orbit after removal of the eyeball due to serious trauma or ocular cancer requiring enucleation [51]. The most commonly used options include hydroxyapatite, polyethylene, or alumina porous spheres, which are “buried” under the patient’s conjunctiva and are postoperatively invaded by fibrovascular tissue [52,53]. These implants are then covered by external polymethylmethacrylate (PMMA) ocular prostheses that are placed over the conjunctiva for aesthetic purposes [54]. 

A severe complication during surgery and in the mid-term is the risk of implant-related infections; during the operation, the porous spheres are typically impregnated with antibiotic solutions that, however, exhibit a burst release and are ineffective against late infections [55]. Baino et al. [56] tested the antibacterial efficacy of composite coatings made up of silver nanoclusters embedded in a silica matrix. These silver/silica coatings can be deposited on the orbital implant surface, as well as on the posterior surface of the PMMA prosthesis in contact with the patient’s conjunctiva. The coatings were mechanically well adherent to the substrate and revealed a potent antibacterial activity inhibiting the growth of *Staphylococcus aureus* in vitro. The antiseptic action was related to the release of silver ions that disrupt the bacterial membrane; interestingly, ion-based therapy was suggested as a promising alternative to antibiotics as metallic ion release does not create the problem of bacterial resistance.

**Figure 2:**
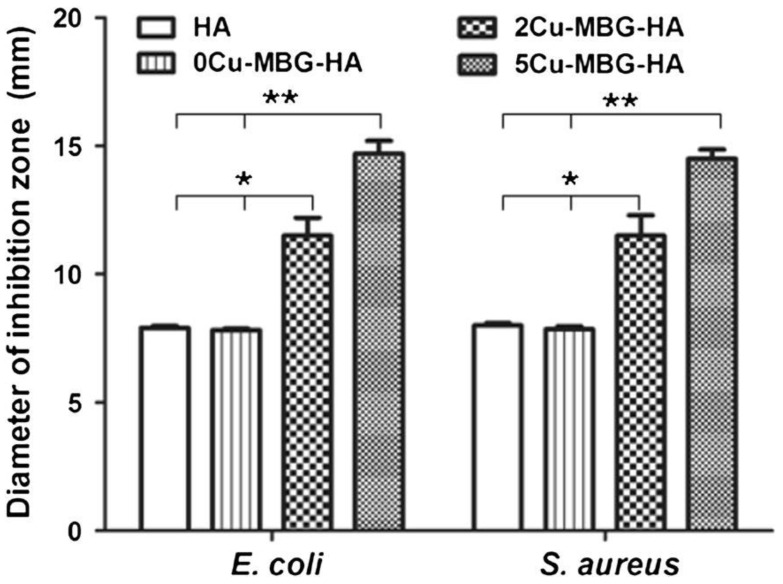
Diameter of the inhibition zones of *Escherichia coli* and *Staphylococcus aureus* growth formed in vitro around the wells (7.8 mm diameter of Oxford cup) filled with hydroxyapatite orbital implants coated or not with the copper-doped mesoporous bioactive glass (MBG) layer (* *p* < 0.05; ** *p* < 0.01). Reproduced from Reference [57].

A similar approach, based on the deposition of an antiseptic layer, was reported by Ye et al. [57] who coated porous hydroxyapatite orbital implants with copper-doped mesoporous bioactive glass (MBG). This study aimed at synergistically combining the antibacterial effect of released copper ions, which are able to kill bacteria via the generation of reactive oxygen species, lipid peroxidation, protein oxidation, and DNA degradation [58], and ofloxacin, an antibiotic hosted inside the glass mesopores (pore size within 3–5 nm). In vitro tests showed that copper-doped implants inhibited the viability of *S. aureus* and *Escherichia coli* (Figure 2); furthermore, since drug loading and release capacity was less efficient in the samples with higher copper concentration, a predominant antiseptic effect of released copper ions over antibiotic molecules was suggested [57].

In addition to eliciting an antibacterial effect, copper ions are known to regulate the expression of many factors involved in angiogenesis, such as VEGF, fibronectin, angiogenin, collagenase, prostaglandin E-1, and ceruloplasmin [59,60].

From a biomolecular viewpoint, copper-induced angiogenesis is thought to be related to the mitogen-activated protein kinase (MAPK) signaling pathway, leading to endothelial cell sprouting [61]. Based on this evidence, Baino [62] first suggested that the copper-doped MBG coating produced by Ye et al. [57] could be useful to promote angiogenesis (fibrovascularization) in porous orbital implants due to the controlled delivery of copper ions. This hypothesis was subsequently proven by Ye’s group who performed primary angiogenic tests in a panniculus carnosus muscle model in rabbits and reported that the copper-doped MBG coating significantly accelerated the vascularization of porous hydroxyapatite orbital implants compared to copper-free materials [63].

Incorporation of copper in glass–ceramic orbital implants was also reported using a nearly inert SiO_2_–CaO–Na_2_O–Al_2_O_3_ glass as a base material [64]. The deposition of a thin copper-doped MBG layer on the walls of glass–ceramic porous implants allowed a sustained release of copper ions to be achieved for potential multifunctional (antibacterial and pro-angiogenetic) action.

To the best of the authors’ knowledge, these are the unique examples of ion-releasing ocular devices. At present, neither commercial orbital implants nor external ocular prostheses provided with inherent antiseptic properties are available on the market.

### 4.2. Microspheres and Nanospheres

Polymeric microspheres and nanospheres are among the most widely used DDSs for the release of biomolecules due to the large availability of different materials that can be used in the transport of therapeutics [65,66]. The size of microspheres is between 1 and 1000 μm while nanospheres are in the range of 10 nm to 1 μm. Nanoparticles are drug transport systems in which the active substance is dissolved or encapsulated or on which it is absorbed or attached. In general, they are small spheres that offer a large specific surface area. Nanocapsules are small tanks with a central cavity surrounded by a polymer membrane in which the drug molecules are dissolved in an oily core or adsorbed at the core/shell interfacial surface. An ideal formulation of nanoparticles allows a prolonged release of drugs and, hence, a long-term therapeutic effect.

Nanosystems are also useful for intravitreal injection because they delay drug clearance, thus reducing the need for repeated injections and the risk of complications. The lipophilic–hydrophilic optimization of the polymer–drug system is a key aspect. The polymers used for the preparation of nanoparticles should exhibit biocompatibility and mucoadhesiveness, swelling properties, adequate molecular weight, a degree of crosslinking, and bioavailability, which depends on the bioadhesion characteristics of the polymer [25].

Synthetic polymers are widely used to synthesize microspheres and nanospheres as they are biocompatible and biodegradable to by-products that can be metabolized by the human body, so that no additional removal surgery is needed [67]. Drug release kinetics should be properly tailored according to the purpose and duration of the therapy by changing the polymer composition. Micro- and nanospheres have the ability to incorporate both hydrophilic and hydrophobic molecules, as well as proteins, peptides, vaccines, and biological macromolecules. If copolymers are used, their characteristics can be adapted for each specific use by modifying the polymer ratios and microstructures and/or by applying strategies of surface functionalization [68].

#### 4.2.1. Poly(lactic-*co*-glycolic) Acid

Poly(lactic-*co*-glycolic) acid (PLGA) is used in many therapeutic devices approved by the Food and Drug Administration (FDA) due to its well-proven biodegradability and biocompatibility [69]. PLGA is obtained by a ring-opening copolymerization reaction of cyclic homodimers (1,4-dioxane-2,5-dions) of glycolic acid and lactic acid.

The catalysts which are commonly used in the synthesis of this polymer include tin 2-ethylhexanoate or aluminum isopropoxide. In the polymerization, the monomeric units (glycolic or lactic acid) are bonded together in the PLGA macromolecules by ester bonds, thus obtaining a linear aliphatic polyester as a product of polymerization.

PLGA is amorphous with a glass transition temperature within 40–60 °C and is soluble in most organic solvents. PLGA properties can be properly tailored depending on the ratio between the monomers; for example, the degradation time (or the persistence) and the mechanical properties can be increased by increasing the amount of lactic acid. Due to its versatility, PLGA is used for making a number of biomaterials, including surgical sutures (lactide:glycolide = 1:9), fixation devices, and biodegradable micro-/nanoparticles for the controlled release of biomolecules such as proteins, small interfering RNAs (siRNAs), and antigens, which can be administered in different ways (intramuscular injections, inhalation, and oral ingestion) [70,71]. Gentile et al. [69] reported that the degradation of PLGA micro- and nanospheres is mainly due to the ratio of lactic acid to glycolic acid. Persistence time can vary from one week to two years depending on the ratio between the co-monomers and the consequent degree of hydrophobicity and crystallinity [72].

The release of drugs from PLGA occurs in a dose-dependent manner and is influenced by the rate of polymer degradation. The release profile often exhibits a three-stage pattern comprising an initial burst followed by a constant release and a final drug burst [73,74]. Furthermore, a smaller particle (from macro- to nanoscale) leads to faster degradation.

PLGA microspheres are mainly used for the release of biomacromolecules such as proteins, deoxyribonucleic acid (DNA), ribonucleic acid (RNA), vaccines, and peptides [75].

PLGA carriers suffer from some limitations, including low to moderate efficiency of drug encapsulation (less than 60% for microspheres and less than 30% for nanospheres), high initial drug burst (20% to 50% of the encapsulated proteins are released in the first 24 h [76]), incomplete release of trapped proteins [54,55], and creation of an acidic environment as a result of the degradation of the hydrophobic part of the polymer. Low values of pH can lead to denaturation and aggregation of proteins, as well as protein instability and, hence, incomplete release [77,78,79,80].

#### 4.2.2. PLGA/Polyethylene Glycol (PEG) Copolymers

Block copolymers of PLGA/polyethylene glycol (PEG) were developed in order to improve the drug release capability of the PLGA microspheres. PEG is a polymer of ethylene glycol that is synthesized via acid or basic catalysis. According to its molecular weight, PEG can be either liquid (low molecular weight) or solid (molecular weight up to 10^6^ Da) in the form of a crystalline polymer [81].

PEGs are biocompatible and are excreted from the body without being modified (they are not very biodegradable); their clinical use is approved by FDA.

PEG carriers form a protective barrier around the drugs, enhancing the biomolecule durability and stability. However, PEG was shown to have a low encapsulation efficiency of antibiotics and proteins, thereby being suitable only for incorporating a limited set of biomolecules [73].

#### 4.2.3. Copolymers with Gallic Acid

In the context of biomaterials for ocular DDSs, gallic acid (GA) was shown to play a very important role in the in situ gelification of gelatin-*G*-poly(*N*-isopropylacrylamide) (GN) copolymers, which combine biodegradable gelatin and thermally reactive poly(*N*-isopropylacrylamide) and are typically used for the intracameral delivery of pilocarpine in glaucomatous patients [82]. Furthermore, GA is known to have antioxidant activity [83]. Chou et al. [82] grafted GA on GN copolymers at various redox reaction times by using a redox pair of ascorbic acid (AA) and hydrogen peroxide as a radical initiator. By increasing the redox reaction time, the total antioxidant activity and the reactive oxygen species (ROS) scavenging capacity against 2,2-diphenyl-1-picrylhydrazyl (DPPH) radicals increased, as a result of the higher amount of GA grafted on the GN copolymers. The physico-chemical properties of GNGA hydrogels were affected by the hydrophilic nature of the GA molecules; specifically, the higher water-absorbing capacity and degradability were directly related to the presence of GA grafts. Pilocarpine encapsulation also depended on the amount of GA grafts, and in vitro drug release studies suggested a three-stage release mechanism, which includes initial burst, diffusion, and polymer degradation. Injections of pilocarpine-loaded GNGA biomaterials into the anterior chamber of glaucomatous rabbits led to a decrease of intraocular pressure (IOP) and pupil diameter. At the end of the in vivo experiments, examination of corneal endothelium revealed that the morphology of hexagonal cells was preserved, thus indicating the safety of the treatment. Biochemical analyses of the rabbit’s aqueous humor also showed that increasing GA graft amounts increased the total level of antioxidants and decreased the overall level of nitrite. Therefore, the potential suitability of GNGA antioxidant hydrogels for the treatment of glaucoma was demonstrated; the grafting of small antioxidant molecules (GA) facilitated proper changes of the physico-chemical properties, drug release behavior and bioactivity of carrier materials.

#### 4.2.4. Copolymers with Polysaccharides

Poly(*N*-acetyl-1,4-β-d-glucopiranosamina), commonly known as chitin, is a biodegradable polysaccharide that can be combined with PLGA to modify and improve the functional characteristics of the polymeric microspheres, such as degradation and drug release kinetics. The cleavage of the β-glycosidic bond between the d-acetylglucosamine unit (chitin degradation) leads to faster weight loss than PLGA degradation [67,72]. Chitin is more hydrophilic than PLGA; thus, degradation is accelerated. The combined PLGA–chitin microspheres produce a particular release pattern with an increase of the initial release rate of the drug followed by a prolonged period (several days) of slow and constant delivery. Mi et al. reported the effect of chitin on the swelling ratio, which indirectly reflects the crosslinking density of the polymer and the rate of degradation that is faster with a decreasing content of chitin [67].

Alginate is another biodegradable polysaccharide that is used for the encapsulation and release of a variety of biological agents, cells, DNA, and enzymes without altering their biological activity [84]. The incorporation of alginate into polymer-based microspheres was shown to reduce drug release discharges (by 1/6 and 1/3 if modified with 1.5% and 0.75% of alginate solution) [85]. Jay and Salzman [86] highlighted how alginate increases the bioactivity and stability of the pro-angiogenic protein VEGF. In a comparative study, Zheng et al. proposed the use of alginate–chitosan microspheres, two hydrophilic protectors, to reduce the acidity of the environment that causes protein denaturation when PLGA microspheres (used as a reference DDS) degrade [78]. Furthermore, this copolymeric system demonstrated a reduced initial burst release (about 20%) and a higher encapsulation efficiency of bovine serum albumin compared to PLGA alone [78].

#### 4.2.5. Gelatin

**Figure 3:**
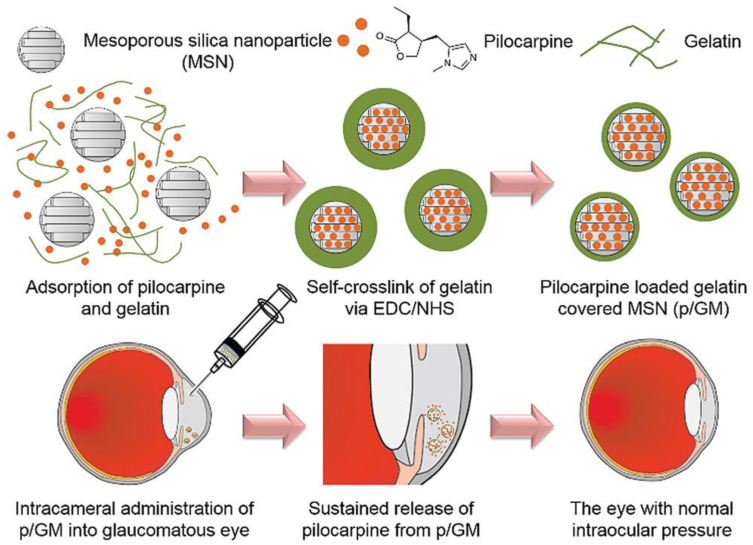
Schematic illustration showing synthesis of silica nanoparticles covered by pilocarpine-loaded gelatin (p/GM) as an ocular drug delivery system (DDS) to decrease the intraocular pressure (IOP) through intracameral injection into the anterior chamber of rabbits’ eye. Reproduced with permission from Reference [94].

Gelatin is a denatured protein derived from collagen (a constituent of the corneal tissue) by acid or alkaline hydrolysis. The applicability of gelatin nanoparticles as ocular DDSs was investigated, and the reported outcomes show promise. Moreover, gelatin can be applied in different forms (e.g., hydrogels) for the repair of various ocular diseases such as focal corneal wounds [87]. Gelatin nanoparticles as a DDS show merits including appropriate compatibility to ocular compartments, very low antigenicity, good mucoadhesive properties, good stability, effective lowering of the IOP, high drug bioavailability, and a lack of irritation [88,89,90]. In 2016, Mahor et al. reported the use of cationic gelatin nanoparticles loaded with moxifloxacin as an effective ocular DDS in the corneal layer [91]. The prepared nanoformulations showed a controlled release of drug up to 12 h and potent antibacterial activity against *Staphylococcus aureus* and *Bacillus subtilis*. The use of hybrid systems based on gelatin was also suggested for effective ocular topical administration of drugs [92]; gelatin nanoparticles loaded with timolol maleate were successfully included in a hydroxypropyl methylcellulose (HPMC) carrier with the aim of reducing the high IOP in glaucoma [93]. In another work, mesoporous silica nanoparticles were covered by pilocarpine-loaded gelatin (p/GM) and intracamerally injected into the anterior chamber of rabbit eyes to extend the drug release time, improve ocular bioavailability, and reduce the IOP (Figure 3) [94]. A high release percentage (50%) with a long-lasting release profile (36 days) was observed in vitro; furthermore, in vivo data showed a successively reduction of IOP. Looking at the existing literature, there is a relative paucity of studies using gelatin nanoparticle-based DDSs for the ocular tissue, and researchers are encouraged to focus more on them. 

#### 4.2.6. Chitosan

Chitosan is a natural polysaccharide copolymer of chitin from crustacean shells, which is structurally composed of glucosamine and *N*-acetylglucosamine units [95]. To date, chitosan is used for various applications in ophthalmology, including contact lenses, solutions, coated nanocapsules, and micro/nanoparticles [96,97]. Chitosan showed the ability to accelerate corneal wound healing via inducing the migration of keratinocytes [98,99]. With regard to drug delivery applications, chitosan offers some advantages including its favorable production cost–benefit ratio, biocompatibility, and biodegradability. Specifically, anti-bacterial and antifungal activities, high mucoadhesiveness, the ability to penetrate the corneal surface (via opening the tight junctions), and excellent ocular tolerance make chitosan a suitable candidate for ophthalmic drug delivery [100,101]. Modified or un-modified chitosan nanoparticles are widely used for delivery of a broad range of therapeutics (e.g., anti-inflammatory, anti-bacteria, and anti-glaucoma drugs) for managing ocular diseases [102,103,104]. In this regard, Li et al. used trimethyl chitosan (TMC)-coated lipid nanoparticles (LPNs) for improving the ocular bioavailability of baicalein (BAI) [105]. The prepared formulation (TMC–BAI–LNPs) had a particle size of 162.8 nm and a positive surface charge (zeta potential of 26.6 mV) with 90.65% drug entrapment efficiency. The drug-loaded nanoparticles showed no ocular irritation in an animal model (rabbits), and the area under the plasma concentration time curve (AUC) was 3.17-fold higher for TMC–BAI–LNPs as compared to controls. The authors concluded that this system could overcome the limited ocular bioavailability of BAI. It should be highlighted that there are several experimental studies in which chitosan nanoparticles were used for ocular drug delivery in combination with other polymers (PLGA, hyaluronan, and alginate) [106,107,108], polysaccharides (cyclodextrin and dextran) [109,110], and lipid mixtures (e.g., lecithin) [111].

#### 4.2.7. Other Polysaccharides

Polysaccharides including cellulose, alginate, pectin, and xanthan gum were previously reviewed in the context of ocular drug delivery approaches [112]. Cellulose and its derivatives are recognized as the first members of polysaccharides used in ophthalmology as topical ophthalmic dosage forms [113]. Due to the water insolubility of pure cellulose, some of its derivatives (e.g., hydroxypropyl methylcellulose (HPMC) and carboxy methyl cellulose (CMC)) are extensively used in treating ocular-related diseases, for example, as eye drops [114,115]. Theses derivatives could also be combined with other polymeric materials for potential use in ophthalmology [116,117]. It was pointed out that cellulose-based macromolecules show the capability of improving the viscosity of formulations and, thereby, may enhance the corneal residence time [118]. In 2019, Orasugh et al. assessed the effect of cellulose nanocrystals (CNC) on drug loading efficacy of triblock poloxamer 407 copolymer (PM)-based in situ hydrogels [119]. The authors aimed to attain a longer pre-corneal residence time and good bioavailability of pilocarpine hydrochloride. For this purpose, a series of composite formulations were developed including those without CNC (called M1) and others containing 0.8%, 1.0%, and 1.2% (*w*/*v*) CNC (called M2, M3, and M4, respectively). The results showed an increase in gel strength along with the sustained release of pilocarpine hydrochloride after addition of CNCs. Furthermore, the cumulative percentages of pilocarpine hydrochloride release from the composites M1, M2, M3, and M4 were 87.26%, 52.89%, 40.43%, and 34.57%, respectively, at 420 min post-test, thus revealing the key role of CNCs in modulating the drug release kinetics. 

**Figure 4:**
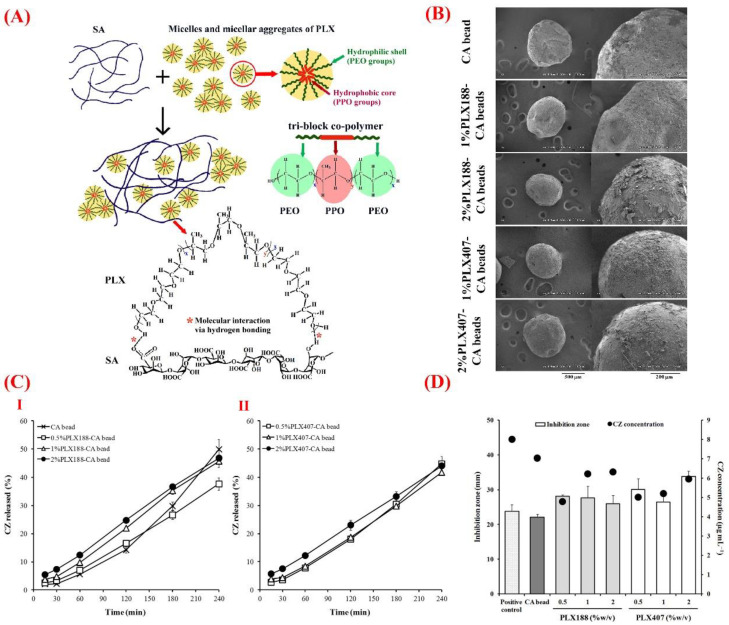
(**A**) Schematic illustration of the molecular interaction between sodium alginate (SA) and poloxamer (PLX), in which carboxyl and hydroxyl groups of SA via hydrogen bonding interact with the hydroxyl groups of PLX. (**B**) Scanning electron microscopy (SEM) showing the surface morphology of calcium alginate (CA) and PLX–CA beads containing clotrimazole (CZ). (**C**) The release profile of CZ from (I) CA and PLX188–CA beads and (II) PLX407–CA beads. (**D**) The graph exhibiting anticandidal activity of CZ released from CA and PLX–CA beads. Reproduced with permission from Reference [125].

Alginate is another natural polysaccharide made of linear unbranched copolymers (1–4)-linked β-d-mannuronic acid and (1–4)-linked α-l-guluronic acid. Biocompatibility, biodegradability, and ease of chemical modification make alginate a suitable vehicle for delivery of a wide range of bioactive molecules such as those used in ocular tissue repair [120,121]. Improved ocular bioavailability of various types of drugs (e.g., lutein) is achievable via their administration by alginate and its composites [122]. Moreover, the use of alginate composites was successfully reported in the context of controlled drug delivery for treating ophthalmological diseases and disorders, such as the posterior segment ocular diseases [123,124]. In this regard, Khlibsuwan et al. recently reported the effectiveness of anticandidal activity of clotrimazole (CZ) loaded in calcium alginate (CA)–poloxamer (PLX) beads [125]. For this purpose, blends of PLX188 or PLX407 into sodium alginate (SA) were added to form dispersions and films and to characterize the PLX–CA beads for increasing the ocular bioavailability of CZ and improving anticandidal delivery. The authors showed that the addition of 0.5% or 1% *w*/*v* PLX (PLX188 and PLX407) to CA could increase the entrapment efficiency of CZ in the beads. Moreover, PLX adding resulted in an enhanced release of CX from the beads and subsequent better activity against *Candida albicans* as compared to CA beads (Figure 4). 

Pectin is a complex renewable carbohydrate polymer found in the cell walls of plants and is mainly formed of galacturonic acid units joined by α-(1→4) linkages [126]. Due to the high gelling capacity, pectin and its composites are widely used in tissue engineering and drug delivery applications [127,128]. The usefulness of pectin in the treatment of ocular tissue diseases was previously documented; for instance, Chan et al. prepared electrospun nanofibers made of pectin–polyhydroxybutyrate (pec-PHB) blended with PHB for retinal tissue engineering and showed that this construct was a suitable substrate for human retinal pigmented epithelium (ARPE-19) cells [129]. In order to further explore the potential of pectin in ocular drug delivery, thiolated pectin nanoparticles were also synthesized by using magnesium chloride as the ionic cross-linker [130]. The obtained data indicated that thiolated pectin (0.01% *w*/*v*) and magnesium chloride (0.01% *w*/*v*) led to achieving 237-nm particles and 94.6% entrapment of timolol maleate as the model drug. Higher corneal permeation of timolol maleate was observed in an ex vivo model of the excised goat cornea by administration of thiolated pectin nanoparticles in comparison to the conventional solution dosage form. In another experimental study, Dubey et al. prepared brinzolamide-loaded pectin–chitosan mucoadhesive nanocapsules via the polyelectrolyte complex coacervation method for potential use as DDS in the management of glaucoma induced in a rabbit eye model [131]. The particle size of pectin–chitosan was in the range of 217.01 ± 0.21 to 240.05 ± 0.08 nm. The results of the in vitro release study showed early burst release and subsequently sustained release of the drug for 8 h, which was significantly better than a marketed formulation. The ex vivo corneal permeation test revealed that the drug-loaded particles were able to cross through the cornea in a higher rate than the marketed product, resulting in a greater IOP-lowering effect. Based on these results, the authors stated that this nano-formulation could be a feasible evolution of conventional eye drops, thanks to its capability of improving the bioavailability (via longer precorneal retention time) and providing sustained release of the drug. It should be highlighted that the potential of pectin as a stabilizer for liposomal drug delivery systems was also documented [132]. 

Xanthan gum is a negatively charged polysaccharide with interesting rheological and gelling characteristics, and it was proposed for ophthalmic applications such as sustained drug release [133,134]. This polymer is also used as an additive to increase the drug release time in comparison to conventional eye drops [135]. Biocompatibility assessments revealed that a fixed combination of 0.09% xanthan gum and 0.1% chondroitin sulfate had no adverse effects on the anterior and posterior segments of a rabbit model for 15 days [136]. In 2014, Amico et al. reported the anti-oxidant effect of 0.2% xanthan gum in human corneal epithelial cells (HCE), as it was able to reduce the level of ROSs to negative control values [137].

### 4.3. Liposomes

The term “liposome” derives from the Greek words “lypos” (fat) and “soma” (body), as liposomes are composed of a lipid double layer, which is similar to a the phospholipidic cell membrane, and cholesterol that surrounds an aqueous compartment [138]. If intravenously injected into an organism, liposomes are conglobated by the cells of the reticuloendothelial system that degrades them, allowing the release of the internal substances.

Therefore, liposomes can be used as vehicles for carrying many drugs and molecules, including proteins, nucleotides, and plasmids, which can be encapsulated in this aqueous compartment [139]. The liposome membrane undergoes deformation under external loads without interrupting its chemical or mechanical properties, thus allowing injection through small-size needles [138,139,140]. Fahmy et al. [141] recently demonstrated the efficacy of anti-glaucoma drugs contained in liposomes that were subconjunctivally injected in an animal model. The diameter of liposomes typically ranges from 50 nm to a few micrometers. Both hydrophilic and hydrophobic drugs can be encapsulated in the aqueous cavity or introduced through the lipid membrane.

Advantages of using liposomes compared to the other drug administration route include the ability of finely controlling the release kinetics, reducing the risk of toxic effects, and extending the drug half-life [138,139,140]; furthermore, liposome exhibit an intrinsic affinity to hydrophobic biological barriers such as the corneal epithelium, thus allowing drug passage.

Modifications of the liposomal surface with other proteins or polymers can provide extra functionalities such as light activation or chemical activation that allow a more targeted and finely controlled release. Verteporfin (Visudyne, Novartis, Switzerland) was the first liposome-based formulation used for the photodynamic therapy of different types of neovascular AMD [142,143,144]. Lajavardi et al. observed an improved topical release of bevacizumab due to the modification of the surface of the phosphatidylserine-based liposome by annexin A5, an anionic calcium-dependent phospholipid binding protein. Furthermore, the same research group used vasoactive intestinal peptide (VIP)-encapsulating liposomes to treat uveitis in rats by intravitreal injection [145].

Limitations of liposomes include the relatively limited amount of transportable drug that can be encapsulated, the need for caution during sterilization procedures, and the risk of interference with vision (blurring effect) after intravitreal injections [146].

### 4.4. Hydrogels

Since their development in the 1960s, hydrogels were increasingly applied in many fields of biomedicine, including DDSs, scaffolding for tissue reconstruction, and cell transplantation [147,148]. The three-dimensional structural network of cross-linked hydrogels protects therapeutic biomolecules from immune reactions and degradation by enzymes. Hydrogels also provide a versatile mean to finely control the drug release kinetics. Furthermore, some hydrogels (e.g., triblock PLGA copolymers) can change their physical properties at different temperatures; in other words, they remain in solution at low temperatures and assume a gelatinous consistency at body temperature [73].

In spite of these potentially attractive features, hydrogels also exhibit several disadvantages including difficult sterilization [149,150], limited shelf life, risk of damage to biopharmaceuticals due to chemical cross-linking reactions [151], risk of toxic effects caused by polymerization initiator residues following polymerization [152], and difficult control of degradation kinetics and drug release due to water absorption during swelling [153].

The use of hydrogels in the ophthalmic field is of great importance for the production of soft contact lenses. In addition to correcting visual defects, hydrogel-based lenses were proposed as local DDSs in order to extend the contact time between drug and ocular surface and minimize drug losses as compared, for example, to topical administration. Studies conducted by Ribeiro et al. [154] led to the synthesis of biomimetic poly(2-hydroxyethylmethacrylate) (pHEMA) hydrogels to be used as carriers of carbonic anhydrase inhibitors for glaucoma treatment. pHEMA hydrogel exhibited a high affinity to these inhibitors and allowed achieving a better control over release behavior. However, further studies are needed to evaluate the long-term biocompatibility and potential interference with the optical and physical properties of soft contact lenses used as a drug carrier [155].

#### 4.4.1. Ionic Force-Sensitive Hydrogels in Topical Administration

Ion-activated hydrogels such as gellan gum or alginate are used to improve the efficiency of topical administration [156]. These hydrogels can be instilled in liquid form in the eye and then undergo gelification in response to pH changes or in the presence of certain ionic species contained in the tear fluid [157]. These gel formulations are able to improve the pre-corneal persistence time of the drug [153]. Regarding the ocular delivery of timolol maleate (TM), Yu et al. could prepare ion-responsive in situ gels containing liposomes by using deacetylated gellan gum (DGG). The produced drug-containing liposomes had a round and uniform shape with a size of below 200 nm. Compared with a routine TM eye drop formulation, the TM-containing liposomes showed a 1.93-fold increase in apparent permeability coefficient, leading to a substantial improvement in the corneal penetration ability. Moreover, a longer retention time on the corneal surface was recorded for the TM-loaded liposome-incorporated ion sensitive in situ gels (TM L-ISG) in comparison with the eye drops. In vivo assessments in rabbit eyes revealed the lack of irritation for ocular tissues. The authors reported the highest efficacy of TM L-ISG at 30 min post-administration, which disappeared after 240 min. In 2015, Kesavan et al. published a report on the usefulness of ion-activated mucoadhesive hydrogels based on gellan or sodium alginate alone and combined with sodium carboxymethylcellulose (NaCMC) regarding the enhancement in the gel bio-adhesion [158]. This formulation showed no irritation in rabbit eyes, without any sign of inflammation. In addition, the results of in vivo antimicrobial evaluation of the muco-adhesive system indicated its efficacy against bacterial keratitis in rabbit eyes. 

There are several pharmacological agents that use hydrogel-based carriers to increase bioavailability. Since these hydrogels can be delivered in the form of liquid eye drops, self-administration by patients is generally easy and well accepted. For example, Ranch et al. could successfully develop a sustained DDS based on a gel of dexamethasone sodium phosphate (DXM) and chloramphenicol (CHL) by using gellan gum in combination with carbopol 940, which could be a viable alternative to conventional eye drops [159].

#### 4.4.2. Thermo-Reactive Hydrogels for Intravitreal Injection

Thermo-reactive hydrogels are recognized as materials capable of swelling or de-swelling in response to the temperature changes of the surrounding fluid. They are commonly categorized into negatively thermosensitive, positively thermosensitive, and thermally reversible gels [160,161]. These materials proved to be effective in the broad field of DDSs and, more specifically, in the context of ocular drug release.

Scientific evidence showed that the main drawback of intravitreal anti-VEGF therapy is the need for repeating the injections every 4–6 weeks over a total treatment duration of two years or more. Thermo-reactive hydrogels are an ideal solution for the localized and prolonged delivery of therapeutic drugs due to their phase transition capacity in response to temperature changes [162,163]. For example, the liquid-to-gel transition temperature of poly(*N*-isopropylacrylamide) (PNIPAAm) is higher than the ambient temperature; thus, this hydrogel is liquid before being injected and then transforms to a gel at body temperature. One of the main drawbacks of PNIPAAm hydrogels is related to the limited amount of drug released in response to temperature changes. In the attempt to address this issue, the use of PEG as a pore-forming agent was suggested to achieve macroporous PNIPAAm hydrogels. For instance, Kang-Mieler et al. developed a thermo-reactive hydrogel by crosslinking PNIPAAm with PEG diacrylate (PEG-DA) or PEG–poly-l-lactic acid (PLLA)-DA through free-radical polymerization [164]. The obtained optically clear hydrogel could be easily injected through small-size needles in the eye at room temperature. Once injected, this copolymer underwent gelification and began to release the encapsulated pharmacological agents [165]. This system was suitable to locally release proteins such as bevacizumab and ranibizumab for about one month and did not induce any long-term side effects on retinal function [166]. It should be stated that applying more highly cross-linked hydrogels could result in smaller pore sizes and longer release times [167]. However, hydrogels having small pore sizes become stiffer and more difficult to inject by small-gauge needles, which makes it difficult to achieve a minimally invasive delivery system to the desired sites (e.g., the vitreous cavity) [164]. Complete hydrogel degradation and drug release could be achieved by incorporating a biodegradable segment in the copolymer [168]. The drug release time could be extended by combining this hydrogel with degradable PLGA microspheres. In order to treat posterior segment diseases, Xie et al. developed an injectable thermosensitive polymeric hydrogel based on PLGA–PEG–PLGA to provide sustained release of Avastin^®^ [169]. The system was prepared by the sol–gel method, and a porous structure (pore size, 100–150 μm) was made. The sustained drug release was recorded over a time of up to 14 days in vitro and no significant toxicity was observed against retinal tissue. A single intravitreal injection of 1.5 mL of 20 wt.% Avastin^®^/PLGA–PEG–PLGA hydrogel (18.75 mg Avastin^®^) in a rat model showed that the hydrogel apparently extended the Avastin^®^ release over time in vivo. 

#### 4.4.3. Cell-Releasing Hydrogels

In addition to releasing pharmacological agents, hydrogels can be used to provide cells for the cell therapy of degenerative retinal diseases. Hydrogel-based cell release systems can play an important role in promoting the survival, differentiation, and integration of delivered cells [170,171]. For example, Ballios et al. developed a temperature-responsive biodegradable hyaluronic acid/methylcellulose injectable hydrogel containing stem cells and this system was able to support the survival, proliferation, and integration of stem cells in the degenerated retina [172].

### 4.5. Combined Systems

A very interesting and effective strategy to overcome the major disadvantages of the various drug release routes described above involves a combination of micro-/nanospheres or liposomes with hydrogels in order to develop a complex, multifunctional system. The benefits obtained using this approach may include a reduction of the initial burst release, a better confinement of carrier/drug in the injection site, and an extension of the drug release time. Furthermore, a higher number of drugs and their combinations can be simultaneously encapsulated in polymeric spheres and hydrogels. Recent studies reported the suitability of PLGA microsphere/PLA–PEG–PLA hydrogel combined system for applications in the treatment of ischemic vascular diseases, cartilage regeneration, and spinal cord injury treatment [173].

In the context of ocular drug delivery, Lajavardi et al. [174] incorporated VIP-loaded liposomes into hyaluronic acid hydrogel for the treatment of intravenously induced uveitis. This combination could increase the drug residence/release time and proved to be effective in reducing eye inflammation. Kang-Mieler et al. recently combined PNIPAAm injectable thermo-reactive hydrogel with PLGA microspheres (ratio of 75:25) to create a microsphere–hydrogel ocular DDS that was able to encapsulate ranibizumab or aflibercept and release both anti-VEGF drugs in a controlled way for about 200 days [175,176]. The drug concentration provided to the retina could be controlled depending on the amount of microspheres suspended in the hydrogel. The efficacy of this system was tested in the treatment of the laser-induced CNV murine model with the aim of replacing the current monthly/bimonthly anti-VEGF therapeutic treatments [177].

## 5. Clinical Applications

A number of ocular DDSs are approved for routine clinical use or are somehow involved in clinical trials; an overview is provided in Table 2.

Some further details about the most challenging applications are given in the sections below.

### 5.1. Glaucoma

Glaucoma is a pathology generally characterized by the increase in IOP, with consequent damage to the optic nerve which can lead to blindness [178]. The pressure increase is often due to the insufficient drainage of the aqueous humor through the physiological pathways. Depending on a number of factors (e.g., stage of the pathology, patient’s co-operation, and general clinical situation), IOP reduction can be obtained through the application of local (eye drops) or systemic hypotonic drugs, laser-based para-surgical treatments, or surgical approaches that aim at improving aqueous humor drainage by the creation of alternative pathways. In recent years, new therapeutic horizons were opened through the use of novel biomaterials and DDSs.

**Figure 5:**
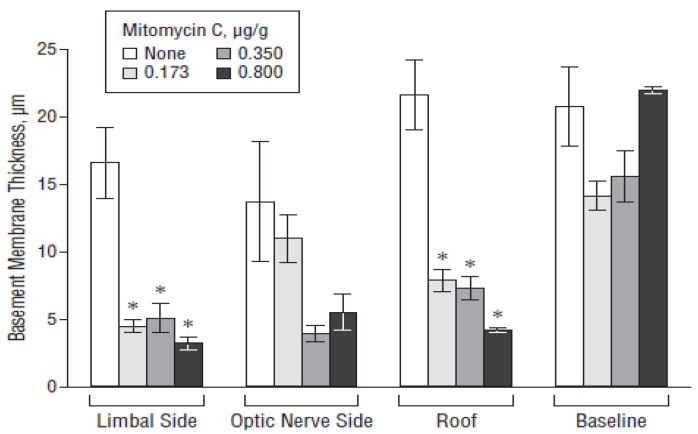
Effect of the poly(2-hydroxyethylmethacrylate) (pHEMA)/mitomycin C-modified Ahmed valve on the fibrous capsule thickness in various regions of the bleb wall. The bar designated as “none” represents the control rabbits implanted with an unmodified Ahmed valve. * *p* < 0.05. Reproduced from Reference [183].

The surgical treatment of glaucoma aims at reducing the IOP values, which can be achieved by implanting drainage devices in the eyeball. The reason behind the high failure rate of this type of surgery is postoperative fibrosis, which occurs after 3–5 years due to excessive scarring in about 30% of cases [179]. Ayala et al. [180,181] studied the effect of various biomaterials on the formation and degree of fibrosis in experimental animal models. Blake et al. [182] pioneered the use of pHEMA hydrogel incorporating the anti-fibrotic drug mitomycin-C. The polymer was first washed in order to remove low-molecular-weight toxic substances (polymerization residues), and mitomycin-C was then introduced into the matrix. pHEMA activated the release of the drug in contact with water; this mechanism is very suitable for glaucoma surgery as the water-based aqueous humor flows to the surgical site where the mitomycin-C is released to reduce fibrosis. The results obtained in vitro using human conjunctival fibroblasts revealed that mitomycin-C inhibited cell proliferation in a dose-dependent way. Sahiner et al. [183] inserted mitomycin-C-loaded pHEMA discs onto Ahmed’s glaucoma valves (AGVs) implanted in rabbits and observed that the drug release occurred upon hydrogel swelling due to contact with aqueous humor. Histological data showed the formation of a thinner fibrous capsule in the animals receiving the DDS, which was also not damaged by ultraviolet (UV) sterilization processes (Figure 5).

Hovakimyan et al. [184] developed a novel glaucoma drainage device incorporating a local DDS to increase safety and efficacy following implantation in the suprachoroidal space. Two different polymers were used in this work, i.e., poly(3-hydroxybutyrate) (P3HB) and poly(4-hydroxybutyrate) (P4HB), in which mitomycin-C or paclitaxel was incorporated. Drug-loaded polymeric films were attached to small silicone tubes to create the DDS-incorporating drainage system. These devices were then implanted in the suprachoroidal space of rabbits, and the IOP was periodically measured in a non-invasive way. After six weeks, the rabbits were sacrificed, and the enucleated eyes were examined by optical coherence tomography of the anterior segment (OCT), magnetic resonance imaging (MRI), and histological assessment. The results confirmed that an effective reduction of the IOP was achieved in vivo; in vitro release of mitomycin-C was faster from both polymers as compared to paclitaxel, while the release from the matrix of P3HB was slower for both drugs. No pronounced fibrosis was observed in any of the groups, but both drugs caused damage to the retina. In order to avoid these negative effects, it will be necessary to use or find new drugs with lower cytotoxicity; furthermore, future studies should be focused on evaluating the long-term efficacy of anti-fibrotic agents.

Implantation of an AGV, which was cleared for clinical use by the FDA in 1993, is perhaps the preferred surgical treatment for glaucoma. Clinical studies reported that the use of AGV is associated with a relatively lower incidence of postoperative complications compared to other aqueous humor drainage devices [185,186]. The success rate of AGV implantation is about 80% one year after surgery, but the five-year success rate drops to 40–50% due to postoperative bleb scarring (i.e., the bleb loses its function). Administration of 5-fluorouracil (5-Fu) is effective to inhibit bleb scarring; as a single application of 5-Fu can reduce postoperative bleb fibrosis only for a limited time period [187], there is the need for multiple local injections that, however, increase the risks of corneal damage, bleb leakage, ocular hypotony, intraocular infection, and other side effects [188]. Therefore, the development of a DDS to be combined with AGV is key to improve the success rate of glaucoma treatment. Bi et al. [189] conducted a study on the possible inhibition of postoperative bleb scarring in rabbit eyes by a (5-Fu)-loaded polycaprolactone (PCL) film attached to AGV. Eighteen New Zealand white rabbits were randomly and evenly divided into three different groups, i.e., group A (combined application of the 5-Fu–PCL prolonged-release film and the AGV), B (local 5-Fu infiltration and AGV implantation), and C (AGV only). Preliminary in vitro tests revealed that the 5-Fu–PCL sustained-release film maintained a release concentration range of 13.7 ± 0.12 to 37.41 ± 0.47 μg/mL over three months. In vivo tests showed the presence of diffuse blebs with ridges in all eyes of group A, two blebs in group B, and no bleb in group C. The postoperative IOP of groups A and C stabilized at 6.33–8.67 mmHg and 7.55–10.02 mmHg, respectively. Histopathological examination showed that the fibrous tissue thickness of the blebs in group A was significantly thinner than that of the other groups, further supporting the suitability of the 5-Fu–PCL DDS in promoting the inhibition of bleb scarring after AGV implantation.

### 5.2. Corneal Transplantation

Immunological allograft rejection is considered the major cause of corneal graft failure following transplantation (keratoplasty) [190]. The exact mechanisms leading to graft rejection are not yet fully understood; it is believed that, with regard to the epithelial and stromal rejection, a vascularized cornea may deliver effector cells to the corneal grafts through the corneal limbus pathway, while, with regard to the endothelial rejection, leukocytes are likely to exit from the iris ciliary body and pass through the anterior chamber before targeting the corneal grafts [191,192]. Therefore, the intraocular immunosuppressive reaction can indeed influence the development of immune reaction and the long-term corneal graft survival.

**Figure 6:**
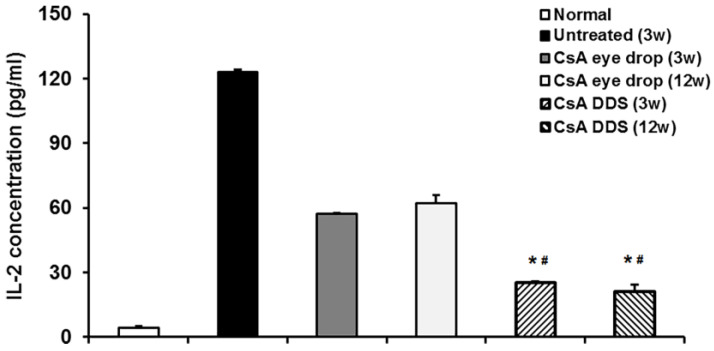
Interleukin-2 (IL-2) level in the aqueous humor of rabbit eyes after keratoplasty. The ELISA test demonstrated that IL-2 concentration significantly increased in the untreated group at three weeks, and cyclosporine–DDS treatment more effectively decreased the level of IL-2 in the aqueous humor as compared to eye drops at all time points. * *p <* 0.01 vs. untreated, # *p <* 0.01 vs. CsA eye drops (*n* = 6 per group). Reproduced from Reference [194].

Shi’s research team reported that PLGA/PCL DDSs releasing cyclosporine, an immunosuppressive drug, were effective in decreasing the rejection rate and prolonging the survival time of corneal allografts in both rabbits and human patients [193,194]. The mechanism involved was thought to be related to the general improvement of the immune microenvironment in the corneal allograft, iris ciliary body, and aqueous humor (Figure 6).

### 5.3. Macular Edema

Retinal pathologies affecting the macular area (maculopathies) impressively increased in the world population over the last few decades due to the increase in people’s average life, which involves an increase of age-related degenerative diseases, as well as metabolic chronic pathologies such as diabetes, which is a major responsible of macular edema and diabetic retinopathy [195].

At present, there are a couple of implantable DDSs (Ozurdex and Iluvien) that are mainly used in the treatment of macular edema.

Ozurdex is a biodegradable implant pre-loaded with 0.7 mg of dexamethasone. It is injected into the vitreous with a 22-gauge applicator without the need for suturing. This DDS is based on a PLGA matrix which slowly degrades into lactic acid and glycolic acid allowing the prolonged release of dexamethasone over a period of six months, without leaving any residues in the eye (the degradation products are water and carbon dioxide). Ozurdex is clinically approved for the treatment of macular edema following retinal vascular occlusion, diabetic macular edema, inflammation of the posterior segment, and uveitis [196,197,198,199].

Iluvien^®^ (Alimera Sciences, Inc., USA) is a multi-polymeric mini-implant for intravitreal injection consisting of a 3.5 mm (length) × 0.37 mm (diameter) polyamide tube containing 190 μg of fluocinolone acetonide (FA) loaded in a poly(vinyl alcohol) (PVA) matrix; one end of the tube is capped with PVA and the other end is sealed with silicone adhesive. The end capped with PVA is permeable to aqueous fluids and controls the release of FA upon hydration of the PVA matrix. This DDS is injected through the pars plana without the need for suturing. It was estimated that 0.2 μg of drug is released every day for about three years; the drug release stabilizes after an initial period. The release constant is slightly higher at the beginning, but in the long term it reaches a stable value. On the basis of clinical studies, this DDS obtained clearance for commercialization in Europe and the United States of America (USA) for the treatment of chronic diabetic macular edema when it is irresponsive to other therapies [200,201].

Apart from Ozurdex and Iluvien, other experimental DDSs were tested for the treatment of macular edema. For example, Prata et al. [202] tested dexamethasone-releasing biodegradable implants based on PCL or PLA prepared by solvent-casting or simple casting. In general, PLA-based devices released only a fraction of the immobilized drug in the first week and, then, drug delivery was negligible. On the contrary, drug was released in a controlled way from PCL-based devices over two months in vitro. The preparation method had little influence on the release profiles. However, simple casting allowed obtaining a more uniform distribution of dexamethasone in the polymer matrix and, therefore, was selected as the prepared fabrication method, also considering that this route was easier and avoided the use of potentially toxic organic solvents. It was also suggested a minimum theoretical drug-to-polymer ratio of 0.25 for PCL-based implants.

Fialho et al. [203] showed that PCL-based implants allow a prolonged release of dexamethasone to be achieved in vitro, as well as a good short-term intraocular tolerance in rabbit eyes. Gaudana et al. [7] also reported a prolonged release of dexamethasone from PCL implants (25% of the drug over 21 weeks). 

### 5.4. Maculopathy

Protein- and peptide-based therapies were recently introduced for the treatment of AMDs. For this purpose, anti-VEGF therapeutics such as pegaptanib (Macugen^®^), ranibizumab (Lucentis^®^), aflibercept (Eylea^®^), and bevacizumab (Avastin^®^) are typically injected intravitreally.

These large molecules have a potent anti-angiogenic effect and low toxicity as compared to smaller molecules, but they are sensitive to physical and chemical degradation, have a short half-life in vivo, and suffer from difficult distribution to the target site. In fact, their therapeutic efficacy is reduced by their high molecular weight and complex structure, which lead to difficult passage through the cell membranes and attack by the cells of the endothelial reticular system [204].

Various in situ gelling materials proved to be potentially safe for use as intravitreal DDSs. Tyagi et al. [205] developed an in situ light-activated gelling system based on PCL dimethacrylate/HEMA that allowed the prolonged release of bevacizumab after being injected into the suprachoroidal space. Wang et al. [206] studied the in vitro release of bevacizumab from a heat-sensitive gel based on poly(2-ethyl-2-oxazoline)–PCL–poly(2-ethyl-2-oxazoline) (PEOz–PCL–PEOz) triblock copolymer which allowed achieving a drug release time of 18 days. PLGA-based hydrogels also showed promise for the intravitreal release of triamcinolone acetonide [207].

## 6. Conclusions

The regulation of ocular cell/tissue response by controlled drug release still remains a challenge. Key aspects to take into account concern both the administration route/DDS used and the drug in itself.

While selecting the DDS material, the placement site should be carefully considered to find out the possible factors accelerating or inhibiting the delivery (e.g., physiological constraints, tissue barriers, blood/fluid flow). Moreover, the inherent physico-chemical properties of the drug to be delivered should be taken into account, including molecular size/weight, charge, and hydrophobicity/hydrophilicity.

If the administration route involves injection, the drug carrier must deform under shear stresses without losing its integrity. An ideal DDS should also be fully biodegradable without leaving any toxic residues in the ocular tissues. Finally, sterilization procedures should not alter the properties of carrier and drug; as this could be tricky for organic materials like pharmaceutics and polymers, such a problem should be somehow considered at the design stage. 

In general, extended drug release by using DDSs is a highly attractive alternative to traditional methods of ocular drug administration (e.g., eye drops) as it allows bypassing ocular tissue barriers, minimizing the need for the patient’s co-operation (which can be sometimes problematic), and decreasing the frequency of surgical interventions (e.g., intravitreal injections). At present, an ideal biomaterial for making DDSs does not exist but each option has advantages and disadvantages with regard to the given application. PLGA is suitable for preparing resorbable micro- and nanospheres and can be variously combined with other polymers, such as polysaccharides, to modulate the degradation rate. Liposomes are deformable and can easily pass through the hydrophobic ocular tissue barriers; furthermore, they can extend the drug half-life. Hydrogels are perhaps the most versatile systems being injectable, stimuli-responsive (e.g., they undergo gelification upon temperature increase), and properly degradable depending on the polymer formulation. Combined DDSs, such as hydrogels embedding micro- or nanospheres, show superiority over single-material DDSs and have the potential to further increase the control on drug release kinetics, extend the total time of release, and improve drug bioavailability. Finally, the possibility of combining or replacing the use of drugs with the therapeutic effects elicited by metallic ions released from biomaterials deserves to be explored in the future, in the attempt of overcoming some drawbacks related to biomolecules (e.g., thermal degradation, bacterial resistance to antibiotics). 

## Figures and Tables

**Table 1:** Components of the eye and their functions.

Component	Function
Cornea	The anterior, transparent part of the eye that covers the pupil and iris. It provides most of the eye’s focusing power (about 2/3).
Ocular chambers	Two compartments that are filled with aqueous fluid. The anterior chamber is the space between the cornea and the iris, whereas the smaller posterior chamber is between the iris and the lens.
Iris	The pigmented membrane that gives the eye its color; it lies between the cornea and the crystalline lens and separates the anterior chamber from the posterior chamber. Its main purpose is to block excess light from entering the eye and to control the iris opening or “pupil” for differing amounts of ambient light.
Aqueous humor	A transparent fluid filling the anterior and posterior chambers. It provides nutrition to the areas lacking blood vessels (i.e., the crystalline lens and the cornea).
Crystalline lens	Transparent, biconvex structure located behind the iris; it is responsible for additional power for focusing light onto the retina.
Tenon’s capsule	External to the sclera, it is a membranous structure that envelopes the extraocular eye muscles, as well as the eyeball and optic nerve.
Sclera	Opaque, fibrous outer tunic of the eye mainly composed of collagen. It holds together the contents of the eye and contains openings and canals for the vessels and nerves entering and exiting the eye.
Vitreous humor	Gel-like substance located in the posterior portion of the eye, filling in the area between the lens and the retina.
Choroid	Rich in blood vessels, provides nutrition to the retina.
Retina	Multilayered sensory tissue of the posterior eyeball onto which light entering the eye is focused, forming a reversed and inverted image. It contains photosensitive receptor cells, the rods and cones, which are capable of converting light into nerve impulses that are conducted and further relayed to the brain via the optic nerve.
Optic nerve	Structure at the back of the eye responsible for carrying nerve impulses from the retina to different areas of the brain.
Conjunctiva	The mucous membrane covering the anterior sclera and the posterior aspect of the eyelids.
Extraocular muscles (EOMs)	Six muscles which control the movement of the eye and are responsible for movements along three different axes: horizontal, vertical, and torsional. Horizontal movements are controlled entirely by the medial and lateral rectus muscles. Vertical movements require the coordinated action of the superior and inferior rectus muscles, as well as the oblique muscles. The oblique muscles are also primarily responsible for torsional movements.

**Table 2:** A short list of ocular drug delivery implants in clinical trials (from a market survey performed in June 2020).

Brand Name	Material	Active Ingredient	Dosage Form	Indication
Vitrasert^®^	PVA, EVA	Ganciclovir	Intravitreal implant	AIDS-related CMV retinitis
Retisert^®^	PVA, silicone	Fluocinolone acetonide	Intravitreal implant	Noninfectious uveitis, posterior uveitis
Ozurdex^®^	PLGA	Dexamethasone	Intravitreal implant	- DME - CRVO - BRVO - Posterior uveitis
Iluvien^®^	Polyimide	Fluocinolone acetonide	Intravitreal implant	- DME - Wet AMD
Yutiq^®^	Polyimide	Fluocinolone acetonide	Intravitreal implant	Chronic noninfectious uveitis
DEXYCU^®^	Acetyl triethyl citrate	Dexamethasone	Intraocular implant	Postoperative inflammation
OTX-TKI/IVT	Hydrogel	TKIs; anti-VEGF	Intravitreal implants	AMD
PDS	Undisclosed polymer	Ranibizumab	Intravitreal implants	Wet AMD
Brimo PS DDS^®^	PLGA	Brimonidine tartrate	Intravitreal implants	- Pars plana vitrectomy AMD - Retinal detachment - Geographic atrophy MD
Rysmon^®^ TG	Methylcellulose	Timolol maleate	Eye drop	Glaucoma
Betoptic S^®^	Amberlite^®^ IRP-69	Betaxolol	Eye drop	Glaucoma
Timoptic-XE^®^	Gellan gum	Timolol maleate	Eye drop	Glaucoma
AzaSite^®^	Polycarbophil	Azithromycin	Eye drop	Bacterial conjunctivitis
AzaSite Plus™	Polycarbophil	Azithromycin/Dexamethasone (ISV-502)	Eye drop	Blepharoconjunctivitis
Lumitect™	Silicone matrix	Cyclosporin	Episcleral implant	GVHD and corneal allograft rejection
I-vation™ TA	PMMA/EVA	Triamcinolone acetonide	Intravitreal implants	DME
Visudyne^®^	Liposome	Verteporfin	Intravenous injection	Wet AMD
Durezol™	Emulsion	Difluprednate	Eye drop	DME
Cortiject^®^	Emulsion	Corticosteroid prodrug (NOVA-63035)	Intravitreal injection	DME
Surodex™	PLGA, HPMC	Dexamethasone	Subconjunctival implants	Postoperative inflammation following cataract surgery
Lacrisert^®^	HPC	HPC	Cul-de-sac implants	Moderate to severe dry eye syndrome, including keratitis sicca
Murocel^®^	Methylcellulose (MC)	PEG, PVA, Povidone	Eye drop	Dried eyes
Celluvisc^®^	CMC sodium	Carmellose sodium	Eye drop	Dried eyes
Ultra Tears^®^	HPMC	PEG, PVA, Povidone	Eye drop	Dried and irritated eyes

AIDS: acquired immune deficiency syndrome, AMD: age-related macular degeneration, BRVO: branch retinal vein occlusion, CMC: carboxy methyl cellulose, CMV: cytomegalovirus, CRVO: central retinal vein occlusion, DME: diabetic macular edema, EVA: ethylene–vinyl acetate copolymer, GVHD: graft versus host diseases, HPC: hydroxypropyl cellulose, HPMC: hydroxypropyl methylcellulose, PLGA: poly(lactide-*co*-glycolide), PMMA: poly(methylmethacrylate), PEG: poly ethylene glycol, PVA: poly(vinyl alcohol), TKI: tyrosine kinase inhibitor, VEGF: vascular endothelial growth factor.
